# Characterization of passive permeability after low intensity focused ultrasound mediated blood–brain barrier disruption in a preclinical model

**DOI:** 10.1186/s12987-022-00369-1

**Published:** 2022-09-08

**Authors:** Tasneem A. Arsiwala, Samuel A. Sprowls, Kathryn E. Blethen, Ross A. Fladeland, Cullen P. Wolford, Brooke N. Kielkowski, Morgan J. Glass, Peng Wang, Olivia Wilson, Jeffrey S. Carpenter, Manish Ranjan, Victor Finomore, Ali Rezai, Paul R. Lockman

**Affiliations:** 1 Department of Pharmaceutical Sciences, School of Pharmacy, West Virginia University, HSC, Morgantown, 1 Medical Center Dr, Morgantown, WV 26506 USA; 2 Rockefeller Neuroscience Institute, West Virginia University, 1 Medical Center Dr, Morgantown, WV 26505 USA; 3 Departments of Neuroscience, Neuroradiology, and Neurosurgery, West Virginia University, 1 Medical Center Dr, Morgantown, WV 26505 USA; 4 Department of Cardiovascular and Metabolic Sciences, Lerner Research Institute, Cleveland Clinic, Cleveland, OH 44106 USA

**Keywords:** Blood–Brain barrier, Focused ultrasound, ExAblate Neuro, Disruption

## Abstract

**Background:**

Systemic drug delivery to the central nervous system is limited by presence of the blood–brain barrier (BBB). Low intensity focused ultrasound (LiFUS) is a non-invasive technique to disrupt the BBB, though there is a lack of understanding of the relationship between LiFUS parameters, such as cavitation dose, time of sonication, microbubble dose, and the time course and magnitude of BBB disruption. Discrepancies in these data arise from experimentation with modified, clinically untranslatable transducers and inconsistent parameters for sonication. In this report, we characterize microbubble and cavitation doses as LiFUS variables as they pertain to the time course and size of BBB opening with a clinical Insightec FUS system.

**Methods:**

Female Nu/Nu athymic mice were exposed to LiFUS using the ExAblate Neuro system (v7.4, Insightec, Haifa, Israel) following target verification with magnetic resonance imaging (MRI). Microbubble and cavitation doses ranged from 4–400 μL/kg, and 0.1–1.5 cavitation dose, respectively. The time course and magnitude of BBB opening was evaluated using fluorescent tracers, ranging in size from 105–10,000 Da, administered intravenously at different times pre- or post-LiFUS. Quantitative autoradiography and fluorescence microscopy were used to quantify tracer accumulation in brain.

**Results:**

We observed a microbubble and cavitation dose dependent increase in tracer uptake within brain after LiFUS. Tracer accumulation was size dependent, with ^14^C-AIB (100 Da) accumulating to a greater degree than larger markers (~ 625 Da–10 kDa). Our data suggest opening of the BBB via LiFUS is time dependent and biphasic. Accumulation of solutes was highest when administered prior to LiFUS mediated disruption (2–fivefold increases), but was also significantly elevated at 6 h post treatment for both ^14^C-AIB and Texas Red.

**Conclusion:**

The magnitude of LiFUS mediated BBB opening correlates with concentration of microbubbles, cavitation dose as well as time of tracer administration post-sonication. These data help define the window of maximal BBB opening and applicable sonication parameters on a clinically translatable and commercially available FUS system that can be used to improve passive permeability and accumulation of therapeutics targeting the brain.

## Background

Drug delivery to the central nervous system is a significant hurdle for treatment of most CNS diseases [1]. The blood–brain barrier (BBB) is highly regulated by endothelia critical to neuroprotection and homeostatic control of the brain [2]. It also restricts permeability of most therapeutics into brain. The BBB is composed of endothelial cells, basal laminae, pericytes, and astrocytic foot processes which work together to form a selectively permeable passive barrier between the blood and brain [3]. There is a paracellular barrier between the endothelial cells which is formed by a complex network of tight junction proteins including occludin, junction adhesion molecules, zonula occludins and claudin-5 [4, 5]. Further, small lipophilic compounds which typically penetrate the barrier are actively effluxed by ATP-binding cassette transporters such as p-glycoprotein and breast cancer resistant protein [6]. Collectively, the BBB efficiently reduces the penetration and residence time of drugs within the brain, limiting therapeutic efficacy in CNS diseases.

Current clinical experimental efforts to increase the diffusion of drugs into the CNS include invasive techniques such as convection-enhanced delivery and disruption of the BBB by osmotic agents, i.e. mannitol [7–10]. These techniques have had some success; however, the invasive nature, associated risks, and limited ability to overcome ABC transporter-mediated efflux have limited their complete clinical application [11, 12]. Alternative non-invasive delivery techniques such as intranasal delivery and use of nano-formulations are limited by the lack of site-specific bio-distribution and low control over drug release [9]. Poor clinical translation of current invasive and non-invasive delivery techniques has led to investigation of novel approaches of drug delivery including low-intensity focused ultrasound (LiFUS). LiFUS is an evolving technique explored to open the BBB and target therapeutics to the brain.

Magnetic resonance image (MRI) guided LiFUS utilizes low energy ultrasonic waves to physically open spaces between the endothelial barrier producing transient BBB disruption [13, 14]. In this technique, intravenously administered microbubbles are used with MRI-guidance to produce local BBB disruption within areas of interest. Typically, microbubbles have a gaseous core, entrapped within a polymeric or lipid shell casing [14]. When targeted with LiFUS, microbubbles oscillate at the endothelium in a process referred to as non-inertial cavitations [14]. Microbubbles reduce the extent of ultrasound energy required to produce a biological effect, thereby reducing potential side effects due to thermal ablation [15]. Early preclinical success in LiFUS assisted BBB disruption has led to use of this technique in multiple pathologies such as Alzheimer’s disease and stroke [16–18]. In addition, exploratory clinical trials in BBB disruption are now being conducted in other illnesses such as stroke, tremor, and brain tumors [19–21]. However, current literature shows heterogeneity in the timing and extent of LiFUS-mediated BBB disruption. Currently the relationship between LiFUS-parameters with magnitude, extent and duration of BBB disruption is not well understood.

One factor contributing to variation in drug permeability is the type of device used for sonication [22, 23]. Early preclinical studies were performed on non-standardized and clinically dissimilar transducer devices, resulting in inconsistent BBB opening [24, 25]. Additionally a disconnect between preclinical and clinical data can be attributed to experimental parameters such as frequency and amplitude which directly influence BBB opening [26–28]. However, in clinical experimentation, the FDA approved ExAblate Neuro instrument uses a combination of these parameters to produce a clinically relevant dose measuring cavitation energy resulting from microbubble oscillation [29, 30]. The prescribed cavitation dose causes the system controller to automatically adjust ‘in-procedure’ acoustic power and produce stable microbubble cavitations. Lastly, many preclinical studies report BBB opening by leakage of contrast enhancing dyes like gadolinium. In such reports, massive increase in BBB permeability may difficult to interpret since MRI sensitivity for contrast agents can cause small increases in gadolinium accumulation to appear as falsely high regional uptake [31].

Herein, we characterize the extent and timing of BBB opening following LiFUS mediated disruption using an FDA approved clinical ultrasound device (ExAblate Neuro) and correlated BBB opening with variable cavitation and microbubble doses. Using differently sized tracers, we studied time course of BBB opening to ascertain the time of maximal opening of the barrier. Our results demonstrate increased tracer accumulation correlates with increased cavitation dose, and increased microbubble dose correlates with increased tracer accumulation plateauing at 200µL/kg. Further, maximal opening of BBB after LiFUS occurs at the time of sonication, despite remaining leaky for up to 8 h post-sonication.

## Methods and materials

### Animals

**Fig. 1:**
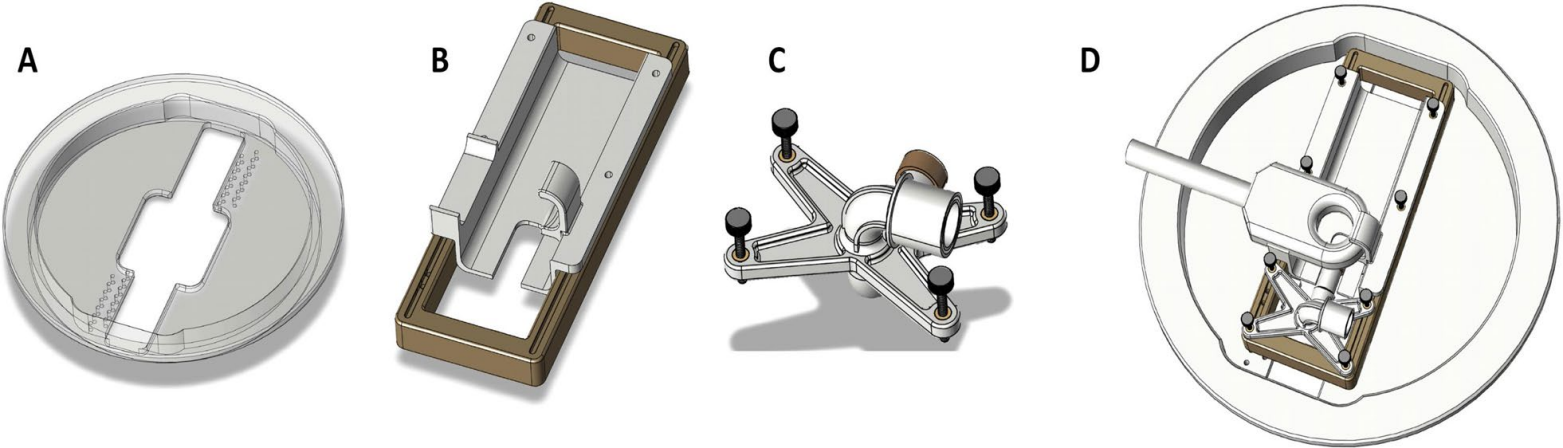
Design of a custom animal restraint for the Neuro-ExAblate. **A** Baseplate to fix the animal restraint over the bowl configuration of the transducer. **B** Custom designed animal bed allowing vertical and horizontal alignment of animal based on size and weight. **C** Anesthesia assembly to allow in-procedure constant anesthesia for rodent holder. **D** Complete rodent set-up for Neuro-ExAblate with fixed coil and animal bed

All experiments were approved by the Institutional Animal Care and Use Committee at West Virginia University. Athymic female Nu/Nu mice were purchased from Charles River Laboratories (Wilmington, MA). All animals were approximately 25 g and 4–6 weeks old at the time of experimentation. Animals were anesthetized using 1.5–2% isoflurane. A plastic frame restraint was created for repeatable placement and adjustment of animals on the ultrasound transducer. The animal holder (Fig. 1) consisted of a baseplate, rodent bed and an anesthesia assembly allowing placement of mice in a supine position with the head partially submerged in degassed water. The transducer base-plate held adjustment dials for vertical and horizontal adjustment based on animal size and weight. A custom nose seal, nose cone extension and adapter were designed on the restraint to allow anesthesia during the procedure. A fixed MRI coil holder was designed to allow repeatable placement of the MRI coil at isocenter. During the experiment, body temperature was maintained with heating pads and mice were monitored for any signs of distress.

### LiFUS experimental design

LiFUS experiments were carried out using the clinical ExAblate Neuro ultrasound technology (InSightec, Haifa, Israel). The transducer (1024 elements, 230 kHz) was placed in the bowl configuration and filled with degassed water. The ExAblate 4000 technology was used in concert with a 3 T clinical MRI machine (Siemens MAGNETOM Prisma). A one channel 3 inch loop coil was used for initial imaging and localization for all experiments. Water was drained for imaging post-sonication.

### Qualitative verification of BBB opening in a healthy mouse model

A T1 Space 3D sequence was used to localize target area and post-contrast imaging. The T1 Weighted Turbo Spin Echo (TSE) sequence variant had FOV = 70 mm in sagittal plane and was reformatted into Coronal and axial planes for targeting. The repetition time was 700 ms, echo time was 7.5 ms, slice thickness was 0.7 mm, frequency & phase encoding matrix = 128 × 128 giving a voxel size of 0.5 mm × 0.5 mm × 0.7 mm, echo train length was 44 (acceleration factor used in TSE imaging for faster scan acquisitions), bandwidth = 434 Hz, number of Excitations (NEX/Averages) = 4 and total Acquisition time was 3:39. Prior to sonication, a bolus of prepared microbubbles (Definity^®^ Lantheus Imaging) was injected intravenously through the tail vein at a dose of 40 μL/kg. Gadavist was injected immediately after the microbubbles. Sonication was started 10 s after injection of the contrast agent. The animals were sonicated at a cavitation dose of 1 for 60 s. In addition to the post-contrast imaging a local macro-hemorrhage sensitive Gradient Echo/Flash weighted sequence was performed with FOV = 200 mm to acquire multi-slices in Sagittal, Axial and Coronal planes. The repetition time (TR) was 7.5 ms, Echo time (TE) = 3.7 ms, Slice thickness = 7 mm, frequency & phase encoding matrix = 256 × 233 giving a voxel size of 0.4 mm × 0.4 mm × 7.0 mm and total acquisition time was 1:10.

### Characterization of LiFUS permeability changes with instrument parameters

#### Microbubble dose

After initial MRI images were acquired, a 1X1 mm spot was localized in the cortical region of the left hemisphere. The effect of microbubble dose was analyzed by intravenous administration of a range of microbubble concentrations of 4, 20, 40, 200, 400 μL/kg through the tail vein. Tracers, including ^14^C-aminoisubutyric acid (^14^C-AIB, 100 μCi/animal, ∼100 Da), Texas Red (TX Red, 6 mg/kg, 625 Da) and 10 kDa dextran (10 mg/kg), were co-administered with microbubbles at the time of injection. Sonication was performed immediately after intravenous administration of microbubbles at 0.3 cavitation dose for 60 s.

#### Cavitation dose

Effect of cavitation dose on BBB disruption was observed at different dose values. Brain imaging and sonication target confirmation were done as stated above. Mice were sonicated using 40 μL/kg Definity for 60 s. Cavitation dose was varied from 0.1 to 1.5 units. The brains were rapidly removed and collected immediately after sonication.

#### Time course of BBB opening

The magnitude of BBB opening was evaluated with tracer administration at different times with respect to the BBB disruption. Sonication was performed at 0.3 cavitation dose for 60 s. Tracers were injected at – 60 s, 1, 3, 6, 8, 12 and 24 h post sonication. Animals received ^14^C-AIB (100 μCi/animal), TX Red (6 mg/kg) and 10 kDa dextran (10 mg/kg). All tracers were allowed to circulate for 10 min post-injection and the brain was collected.

### Brain processing and fluorescence quantification

Following tracer circulation, brains were harvested, flash-frozen in isopentane at − 70 °C. Sectioning was performed using a Leica CM3050 cryostat (Leica Microsystems, Los Angeles, CA) to obtain 20 µm thick slices. Data were analyzed using an Olympus MVX10 fluorescent, stereomicroscope (optical zoom range 0.63–12.6, N.A = 0.5). Sections were imaged using RFP (588 nm), and DAPI (461 nm) channels to detect Texas red (TxRed) and cascade blue (10 kDa dextran) respectively. The same sections were placed in quantitative autoradiography cassettes (GE Healthcare) with corresponding ^14^C standards (0.1–862 nCi/g). A phosphor screen was placed over the samples (Fujifilm Life Sciences, 20 × 40 super-resolution) and slides were allowed to develop for 21 days. Screens were read on a high-resolution phosphor imager (FUJI FLA-7000, Fujifilm, Life Sciences). Permeability of ^14^C-AIB was analyzed using MCID Analysis (InterFocus Imaging LTD). The same slides were then stained with 0.1% Cresyl violet and imaged for histopathological visualization.

### Data analysis

GraphPad^®^ Prism 6.0 (San Diego, CA) was used to determine statistical significance among treatment groups. Statistical significance of the data between two groups was analyzed by the Student t-test (Prism 6). Statistical significance of the data with more than two groups was analyzed by one-way ANOVA with a Tukey post-test (Prism 6). All data represent mean ± SEM unless otherwise indicated (n = 5–7). Based on previous experiments, we anticipated that the sample sizes would provide roughly 80% power to detect a 50% difference between groups. Significance levels were set at p < 0.01 (**), and p < 0.001 (**).

## Results

### Qualitative verification of BBB opening in healthy mice:

To verify BBB opening using the ExAblate Neuro device, we examined distribution of tracers within a single 1 × 1 mm targeted spot within the left hemisphere. Mice were placed on a custom designed animal restraint (Fig. 1) in a supine position. The holder was intentionally designed for repeatable placement of animals within the transducer allowing for accurate, reproducible results (Fig. 1B-D). The skull of each mouse was partially submerged in degassed water within the transducer. Post microbubble administration, Gadavist and tracer injection, animals were sonicated and tracers were allowed to circulate for 10 min prior to euthanasia.

**Fig. 2:**
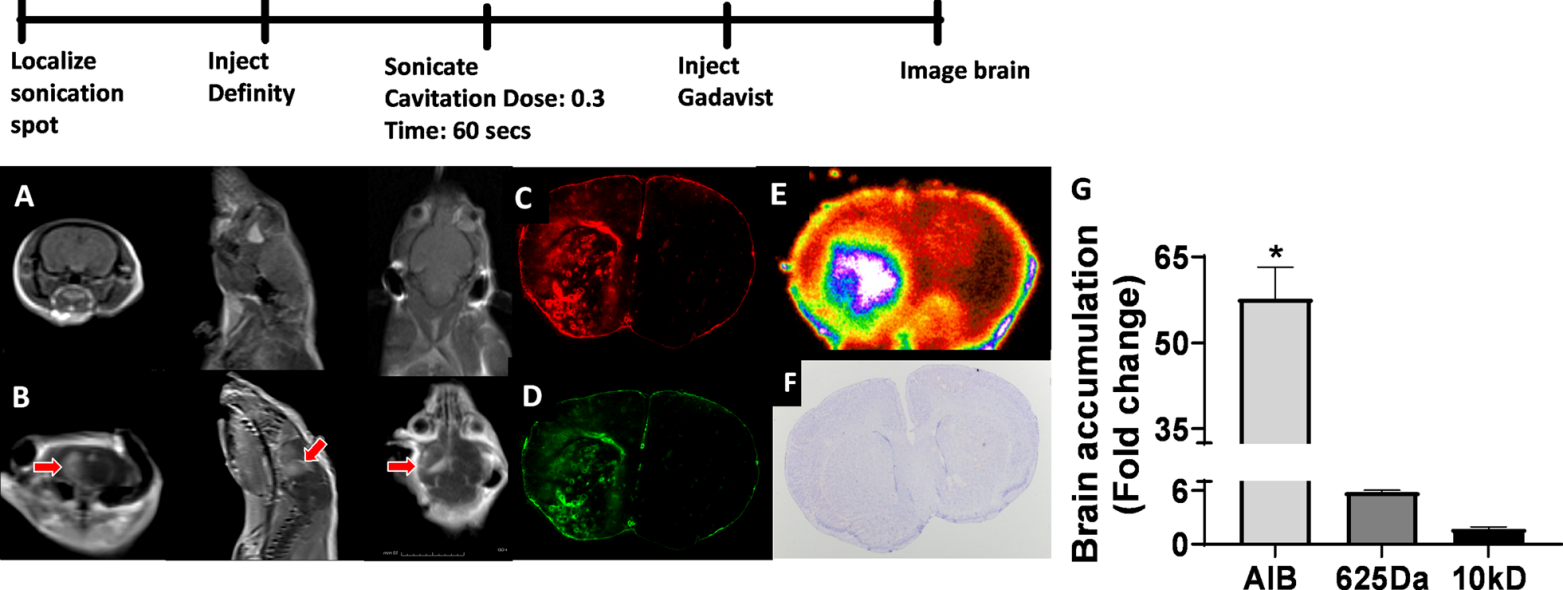
Histological verification of opening of healthy BBB. **A** Coronal, Sagittal and axial MRI images of brain showing localization before sonication. **B** Coronal, Sagittal and axial MRI images showing gadaolinium contrast enhancement in sonicated regions of brain. **C**, **D** Enhancement in intensity of TxRed (625 Da), 10kD dye in sonicated region **E** Autoradiogram showing enhanced accumulation of C-14 aminoisobutyric acid (105 Da) in sonicated region. **F** Cresyl violet staining shows no macroscopic damage to brain. **G** Quantitative analysis shows significantly higher accumulation of AIB, TxRed and 10kD dextran compared to non-sonicated contralateral side (p < 0.01)

We observed gadolinium enhancement within targeted sonicated regions as depicted on T1 MRI in (Fig. 2A, B). Coronal, axial and sagittal planes were evaluated to confirm BBB opening within the sonicated areas (Fig. 2A, B). Fluorescent and phosphorescent imaging (Fig. 2C–E) showed a 58, 5.9, and 1.7-fold increase in ^14^C-AIB, TX Red, 10 kDa dextran accumulation respectively compared to the contralateral, non-sonicated hemisphere (Fig. 2G). Macroscopic evaluation using cresyl violet staining showed no tissue damage (Fig. 2F).

### BBB permeability increases with microbubble dose

**Fig. 3:**
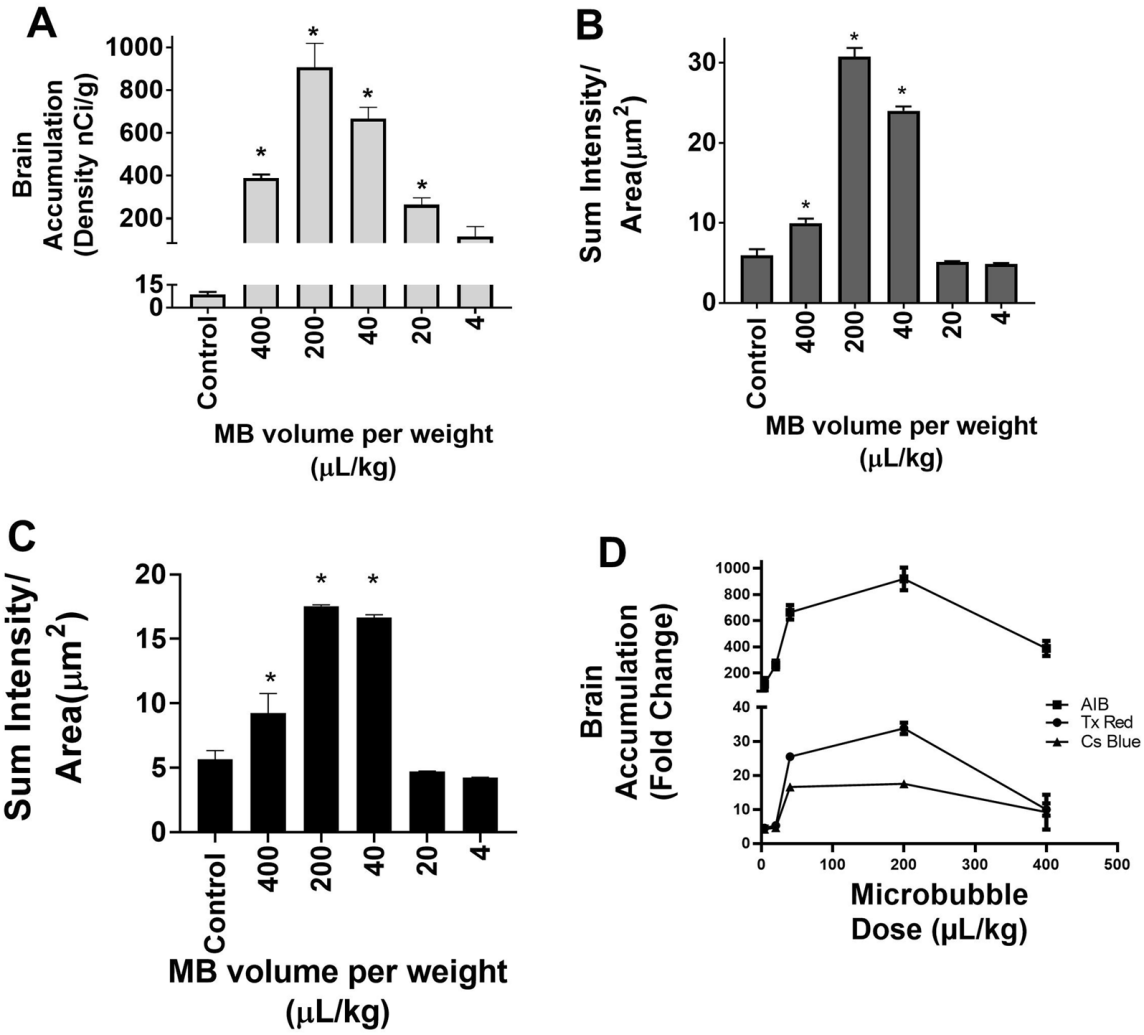
Microbubbles affects tracer accumulation in a dose dependent and size dependent manner: **A**–**C** BBB disruption with LIFU shows significantly higher accumulation of dye for a microbubble dose between 40-200uL/kg for AIB, TxRed and Cs Blue respectively. **D** The accumulation of tracers is size dependent Accumulation C-14 AIB (105 Da) > TxRed(625 Da) > Cs Blue(10kD). (p < 0.01)

Mice were exposed to a range of I.V. microbubble doses ranging from 4–400 μL/ kg. Fluorescent and phosphorescent analysis revealed a dose dependent increase in brain accumulation of each tracer as depicted in (Fig. 3A–C) (p < 0.01). Highest brain accumulation of ^14^C-AIB was approximately 667 ± 53 and 907 ± 112 nCi/g observed at 40 and 200 μL/kg respectively (Fig. 3A). Sum intensity/ µm^2^ reached a near maximum after microbubble injection at a dose of 40 μL/kg for both TX Red, 24 ± 2.3, and 10 kDa dextran, 17 ± 0.9. Peak accumulation of both tracers was observed at a microbubble dose 200 μL/kg (TX Red 31 ± 4.7 sum intensity/μm^2^ and 10 kDa dextran 17.6 ± 0.4 sum intensity /μm^2^). However, this was not significantly different from the accumulation at 40 μL/kg. While concentrations higher than 200 μL/kg showed low tracer uptake, we also observed erythrocyte extravasation observed by cresyl violet and eosinophil staining.

### Increased cavitation dose correlates well with increased tracer accumulation

**Fig. 4:**
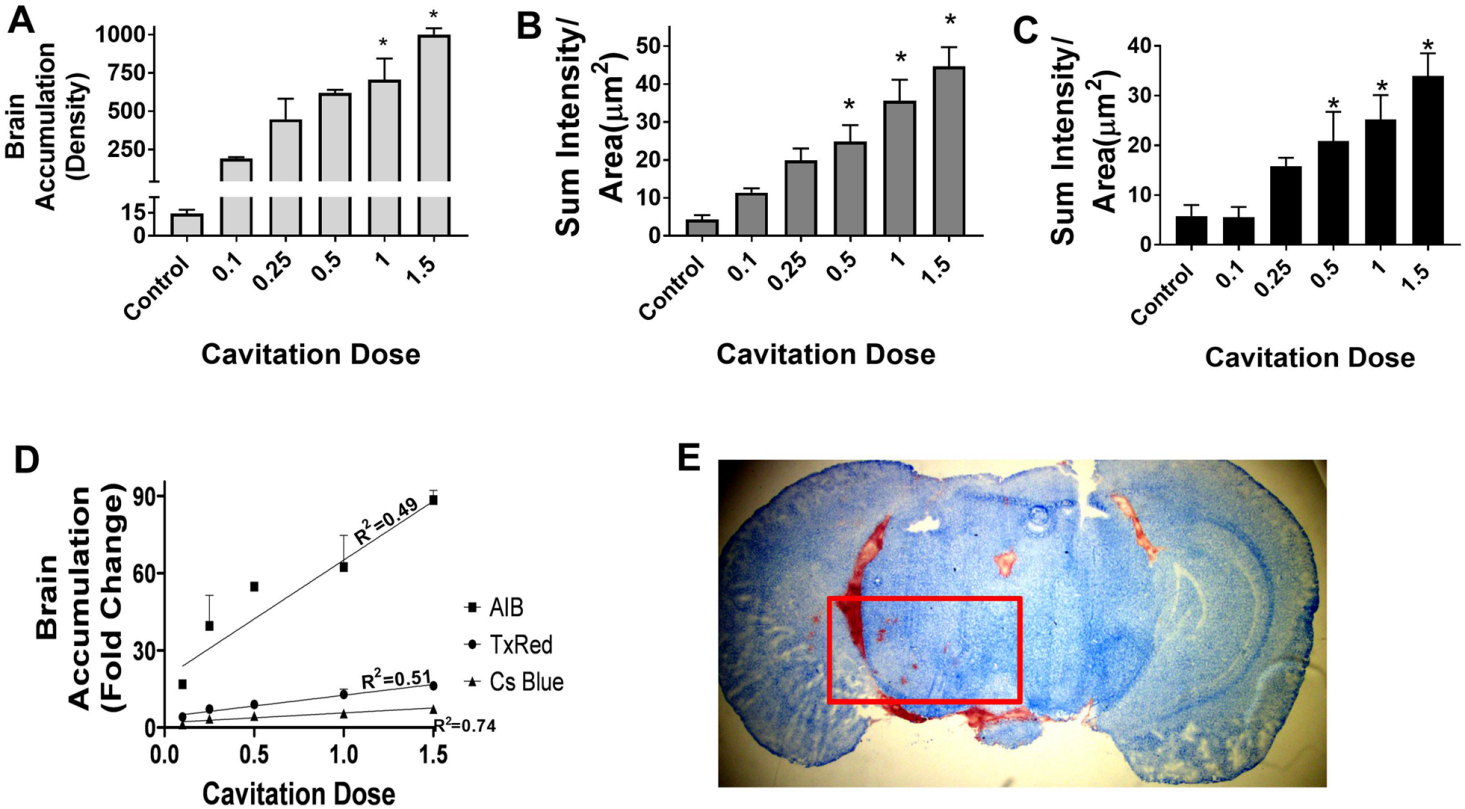
Dose of cavitation affects tracer accumulation in a dose dependent and size dependent manner. **A**-**C** Blood–brain barrier permeability increases significantly with LIFU mediated for doses ranging between 0.1–1.5CD for AIB, TxRed and Cs Blue respectively. **D** The accumulation of tracers is size dependent and shows a linear relation with cavitation dose. **E** Higher cavitation dose above 1CD show erythrocyte extravasation as observed by eosinophil staining (p < 0.01)

The effect of cavitation dose on BBB opening was explored using doses ranging between 0.1 and 1.5. Fluorescent tracers were administered 60 s before sonication and allowed to circulate for 10 min. LiFUS was performed on the left hemisphere with a 40 μL/kg dose of microbubbles and 60 s sonication. We observed a linear increase in marker permeability with increasing cavitation doses as seen in (Fig. 4A–D) (p < 0.01). Control ^14^C-AIB, TX Red and 10 kDa dextran values were 14.5 ± 1.3, 4.4 ± 2.8, and 5.8 ± 2.2 respectively. Further, tracer permeability decreased with tracer size. BBB permeability for ^14^C-AIB, TX Red and 10 kDa dextran was 448 ± 290, 20 ± 7 and 15.9 ± 1.8 at 0.25 cavitation dose. Highest tracer uptake for ^14^C-AIB, TX Red and 10 kDa dextran was 1002 ± 92 density/nCi, 45 ± 13.2 sum intensity/μm^2^ and 33.4 ± 4.5 sum intensity/μm^2^ respectively at 1.5 cavitation dose. However, cavitation doses higher than one showed tissue damage and erythrocyte extravasation with eosin staining (Fig. 4E).

### LiFUS mediated BBB disruption is a transient, time dependent, and predictable process

**Fig. 5:**
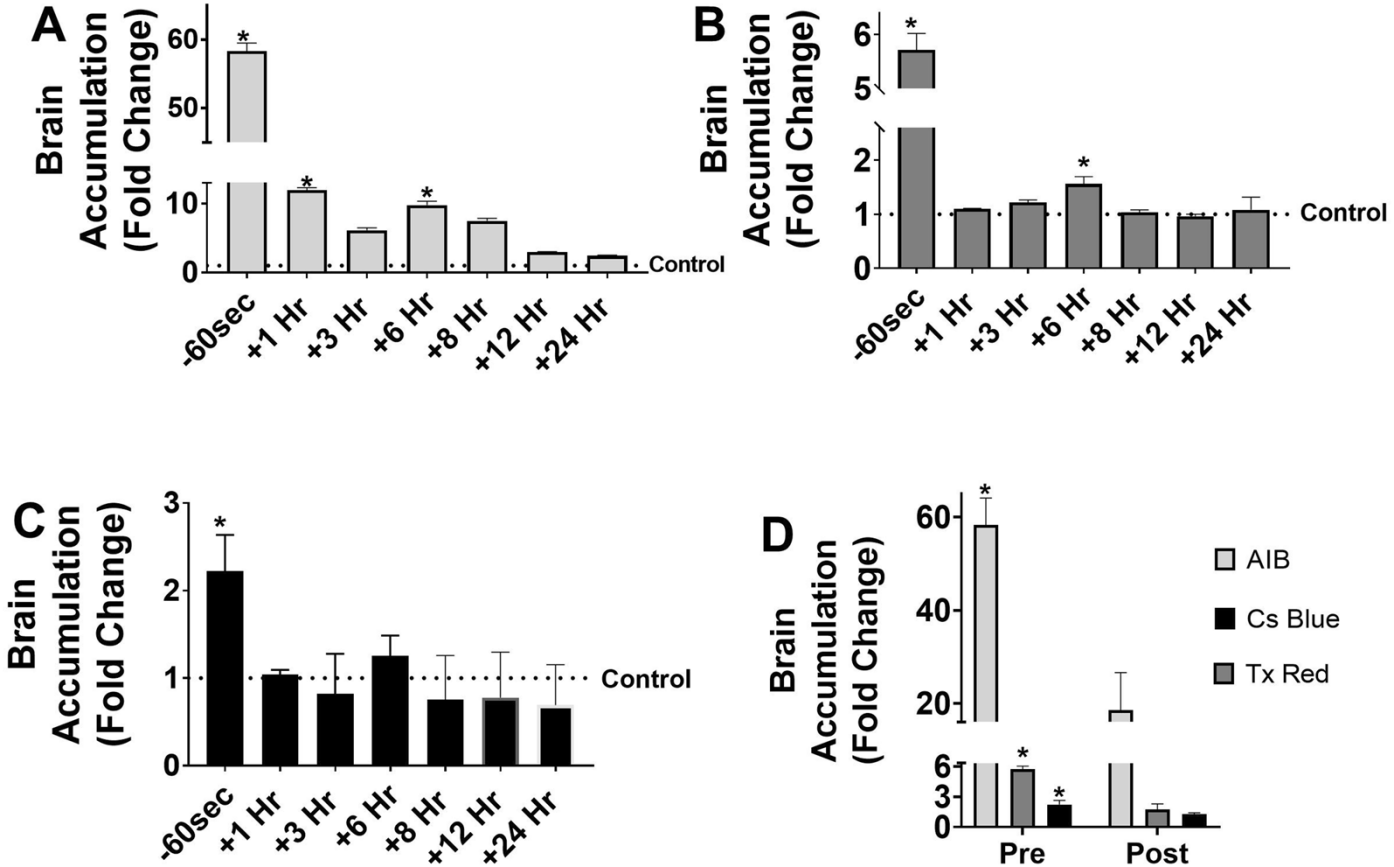
Increase in BBB permeability post LIFU is transient and affects accumulation of drugs/tracers within the brain in a size dependent manner. **A**–**C** Time of drug/ tracer administration post LIFU significantly affects brain accumulation of tracers for AIB, TxRed and Cs Blue respectively. Peak BBB permeability is observed when tracer is administered before sonication or 6-h post-sonication. **D** Significantly higher brain accumulation is observed when tracer is administered on-board or pre-sonication vs when it is administered immediately post sonication. Increase in BBB permeability is influenced by size of administered tracer. (p < 0.01)

We assessed whether time of tracer administration influenced brain accumulation post-LiFUS in mice. Mice were sonicated for 60 s at 0.3 cavitation dose and received tracer 60 s prior to immediately after or 10 min–24 h after sonication. In each case, the tracer was allowed to circulate for 10 min before brain collection. Fluorescent imaging and quantitative autoradiography revealed size dependent permeability for all time points as seen in (Fig. 5). Highest tracer accumulation was observed when tracers were already onboard and within the circulation pre-sonication. Brain uptake for ^14^C-AIB, TX Red and 10 kDa dextran was 58.3, 5.7 and 2.2-fold higher than contralateral hemispheres when tracer was administered before sonication (p < 0.01). Accumulation reduced to approximately 12, 1.7, 1.2-fold compared to contralateral hemisphere for ^14^-C AIB, TX Red and 10 kDa dextran when the tracer was administered immediately after sonication (p < 0.01). We observed a significant increase of tracer permeability at 6 h post-sonication. ^14^C-AIB and TX Red showed approximately 10 and 1.6-fold higher accumulation respectively compared to contralateral hemispheres at 6 h post-sonication (p < 0.01). There was a non-significant increase of 1 and 1.3-fold for 10 kDa dextran post sonication and after 6 h respectively.

## Discussion

Disruption of the BBB by LiFUS is a promising technique to increase delivery of drugs to the CNS. However, the extent of LiFUS-mediated BBB disruption has not been clearly elucidated. This is partly due to discrepancies in the current literature arising from use of varied experimental parameters, different ultrasound delivery devices, and reliance on MRI contrast agents to report extent of BBB opening. In this study, we explored use of a clinical MRI-guided LiFUS (ExAblate) to characterize the extent of opening, size dependence, and time of opening in a healthy model of the BBB. Our results demonstrate a clear tracer size dependent brain accumulation when microbubble and cavitation dose are constant or varied. Increasing microbubble dose linearly increases tracer accumulation within the brain up to 200 μL/kg. Similarly, an increase in cavitation dose ranging between 0.1–1.5 proportionately increased dye uptake within the brain; with larger doses (1–1.5) showing tissue damage. The BBB was observed to be most permeable when tracers were in circulation during sonication (^14^C-AIB, TX Red and 10 kDa dextran was 58.3, 5.7 and 2.2-fold higher than contralateral hemispheres). Interestingly, our results show a significant decrease in tracer accumulation when administered immediately post-sonication. Lastly, we observed a secondary opening of the BBB of a lower magnitude at 6 h post-sonication observed by an increase in uptake of ^14^C-AIB and TX Red. Together, these results highlight how the clinical ExAblate experimental parameters and time of drug administration post-LiFUS can significantly impact passive permeability across the BBB.

Ideally, under control conditions passive permeation across the BBB is achieved when a drug possesses physicochemical properties satisfying Lipinski’s rule of five [32]. In the case of LiFUS-mediated BBB opening, many studies show successful leakage of smaller molecules in the brain post-LiFUS. However, attempts of using LiFUS to deliver larger molecules to the brain, such as immunotherapies has had a lower degree of success [33]. We evaluated magnitude of BBB opening post LiFUS with simultaneous administration of differently sized tracers. Our data demonstrate a clear size dependence of BBB opening post-LiFUS. While LiFUS transiently reduces barrier integrity; the BBB remains functional in limiting passage of the larger tracer. Our report supports previous data evaluating LiFUS-mediated BBB opening in vivo model using an in-house device set-up and Sonovue contrast agent. Similar to our results, the study showed a low-concentration heterogeneous distribution of the higher molecular weight tracer relative to lower molecular weight tracer [34]. The data support the need to optimize experimental parameters to produce sufficient barrier opening for the specific size of the molecule of interest.

A critical point of consideration in LiFUS mediated BBB disruption is dose of microbubbles used prior to sonication. When targeted with LiFUS, microbubbles oscillate at the endothelium in a process referred to as non-inertial cavitations. Force exerted by microbubbles on the tight junctions increases paracellular transport across the BBB. While this is the most common method of passive drug influx, previous research has shown that secondary mechanisms can also upregulate paracellular transport. For example, Deng et. al. found increase in expression of cavelin-1 leading to higher transcellular transport by cavaeole [35]. More recent studies also suggest a decrease in efflux transporters post LiFUS. When combined, these processes can potentially improve drug retention time in the brain and consequently efficacy in the clinic. Previous studies have established a need to optimize microbubble dose to prevent inflammation induced damage to endothelial cells [36]. Preclinical studies typically report microbubble doses ranging from 6 μL/kg to 30 ml/kg [37–40], whereas the clinically recommended dose of Definity^®^ is 4–20 μL/kg [41]. In this experiment, we examined effect of microbubble dosing on passive permeability in our pre-clinical model. Our results suggest that the concentration of microbubbles and tracer size directly influence extent of BBB opening and vascular permeation of tracers. Low tracer uptake is observed at doses higher than 200 μL/kg and at doses lower than 40 μL/kg. Crowding of microbubbles at the targeted region leading to lower oscillation efficiency may be a potential reason for reduced BBB opening at very high doses [42]. On the other hand, low microbubble concentrations cause individual microbubbles to decay quickly with ultrasound exposure, producing insufficient mechanical force for tracer accumulation [43]. We selected 40 μL/kg as a dose for our studies to lower chances of off-target damage while maintaining optimal tracer accumulation. The difference in skull thickness and clearance rates between mice and humans could explain why this dose is slightly higher than the recommended clinical dose. Further, in clinical research, using slow bolus injections or intravenous infusions to cover larger sonication volumes may minimize the decay rate inside the sonication zones [44].

The type of device used for sonication, “individually-designed” or clinically approved, is another key factor for LiFUS-mediated BBB opening. Due to lack of standardized ultrasound devices, previous studies were performed on instruments with unique modifications to allow their use in a pre-clinical setting [24, 25, 45]. The frequency and amplitude produced by these instruments produced variable BBB disruption, making it difficult for clinical translation [46]. For example, Chen et al used passive cavitation detection for a single-element transducer to demonstrate that size-dependent BBB opening for larger molecules was associated with mostly inertial cavitations [47]. In contrast, the FDA approved ExAblate technology monitors applied frequency and amplitude, and automatically adjusts power to maintain effective cavitation dose. The ExAblate Neuro monitors real-time changes in passive cavitations and delivers active energy applied as an acoustic or spectral energy score [29]. If unstable cavitation activity is detected and energy score exceeds applied dose, sonications are automatically stopped [29]. Another factor important for clinical consideration is the frequency used for sonication. In most pre-clinical experiments the frequency used is higher than those used during clinical LiFUS [48]. Clinically, lower frequencies are important to prevent bone attenuation [49, 50]. However, lowering frequency can necessitate use of transducers with high geometric gain, limiting focal area targeted for sonication [48]. This is especially important while targeting areas of interest within a smaller rodent brain. High gain phase array transducers like the ExAblate technology, can limit overheating associated with unpredictable internal wave reflections reducing potential increase in acoustic energies near the skull [40]. Our study established a size dependent increase in BBB opening proportional to cavitation dose applied by the ExAblate. These findings expand on previously reported literature by establishing changes in barrier permeability with size of administered tracer using a clinical device [46, 48, 51]. 

A striking finding within our report is opening and almost immediate resealing of the barrier post LiFUS mediated disruption. Passive permeability of varying sized tracers was significantly higher when tracers were in circulation during sonication as compared to when they were administered within a minute or two after sonication. This may indicate an immediate LiFU induced expansion of tight junctions between endothelial cells followed by instantaneous resealing of majority of the barrier. While the barrier remains leaky for a few hours, it is effective in limiting tracer entry in a size dependent manner (10 kDa dextran > TX Red > ^14^C AIB).

It is notable we observed a lower magnitude second BBB opening at approximately 6 h after LiFU, evidenced by higher penetration of ^14^C-AIB and TX Red in brain. The phenomenon of biphasic BBB opening has previously been reported in several conditions where BBB integrity is damaged including stroke, transient focal ischemia, or traumatic brain injury [52–54]. While an exact reason for this phenomenon is unknown, it is hypothesized primary BBB breakdown leads to activation of secondary neuro-inflammatory pathways which can cause the barrier to be more leaky [37]. While results in this report indicate a lower magnitude of secondary opening, it is possible this response may be elevated in more immunocompetent mice. Lastly, our results highlight size dependent penetration of markers at the time of biphasic opening. Together, these data give insight into windows of a time dependent BBB opening that may be taken advantage of to deliver therapeutics to brain using LiFUS.

Overall, our study delineates impact of LiFUS parameter variation on BBB opening for a range of differently sized drug markers. Further, this work has direct value for translation to understand drug distribution in a clinical setting using the ExAblate Neuro technology. We demonstrated a size dependence of tracer penetration when microbubble dose, cavitation dose and timing of administration are varied using a clinical transducer. Knowledge of these parameters is essential for targeting therapies, especially when the BBB is altered by disease physiology, such as the disrupted brain microvasculature in CNS tumors [2]. Our results identify a potential reason for limited success of large molecule uptake using LiFUS based on dose and time of administration and demonstrate the need for further work to determine ‘large molecule specific’ parameters for LiFUS.

## Conclusion

Combination of LiFUS with intravenously administered microbubbles is a non-invasive technique used to disrupt the BBB. While several studies have established leakage of drugs post-LiFUS using modified devices, we define extent of drug leakage and implication of timed drug delivery. In this study, we document size dependent BBB opening with cavitation dose, microbubble dose and timing of tracer administration. As new clinical trials explore LiFUS dependent therapeutic interventions, it is essential to understand how disease pathology can impact LiFUS mediated increase in drug permeability and therapeutic responses. Optimization of CNS therapeutic regimens would require combination of appropriate LiFUS parameters, knowledge of LiFUS associated changes in brain microenvironment and knowledge of drug pharmacokinetics across the BBB. In addition to BBB opening, the FDA has approved the ExAbate technology for high intensity treatment of tremor and Parkinson's disease, as well as low intensity neuro-modulatory applications [55, 56]. The data presented in this report can potentially serve as a guiding parameter for direct clinical application.

## Data Availability

The datasets used and/or analyzed during the current study are available from the corresponding author, Dr. Paul Lockman on request.

## Abbreviations

BBB: Blood–brain barrier
LiFUS: Low intensity focused ultrasound
MRI: Magnetic Resonance Imaging
TX Red: Texas Red
10 kDa dextran: Cascade Blue
